# Three dimensional cultivation increases chemo- and radioresistance of colorectal cancer cell lines

**DOI:** 10.1371/journal.pone.0244513

**Published:** 2021-01-04

**Authors:** Jana Koch, Dina Mönch, Annika Maaß, Christian Gromoll, Thomas Hehr, Tobias Leibold, Hans J. Schlitt, Marc-H. Dahlke, Philipp Renner

**Affiliations:** 1 Dr. Margarete Fischer-Bosch Institute for Clinical Pharmacology, Stuttgart, Germany; 2 University of Tübingen, Tübingen, Germany; 3 Department of Radiation Therapy, Robert-Bosch-Krankenhaus, Stuttgart, Germany; 4 Department of Radiation Therapy, Marienhospital, Stuttgart, Germany; 5 University of Stuttgart, Stuttgart, Germany; 6 Department of General and Visceral Surgery, Robert-Bosch-Krankenhaus, Stuttgart, Germany; 7 University Medical Centre Regensburg, Regensburg, Germany; Wayne State University, UNITED STATES

## Abstract

Although 2D cell cultures are commonly used to predict therapy response, it has become clear that 3D cultures may better mimic the *in vivo* situation and offer the possibility of tailoring translational clinical approaches. Here, we compared the response of 2D and 3D colorectal cancer (CRC) cell lines to irradiation and chemotherapy. Classic 2D cultures and 3D spheroids of CRC cell lines (CaCo2, Colo205, HCT116, SW480) were thoroughly established, then irradiated with doses of 1, 4, or 10 Gy, using a clinical-grade linear accelerator. The response was assessed by immunohistochemistry, flow cytometry, and TUNEL assays. Upon irradiation, CRC 3D spheroids were morphologically altered. After irradiation with 10 Gy, annexin V/PI staining revealed a 1.8- to 4-fold increase in the apoptosis rate in the 2D cell cultures (95% CI 3.24±0.96), and a 1.5- to 2.4-fold increase in the 3D spheroids (95% CI 1.56±0.41). Irradiation with 1 Gy caused 3- and 4-fold increases in TUNEL positive cells in the CaCo2 and HCT116 (p = 0.01) 2D cultures, respectively, compared with a 2-fold increase in the 3D spheroids. Furthermore, the 2D and 3D cultures responded differently to chemotherapy; the 3D cultures were more resistant to 5-FU and cisplatin, but not to doxorubicin and mitomycin C, than the 2D cultures. Taken together, CRC cells cultured as 3D spheroids displayed markedly higher resistance to irradiation therapy and selected chemotherapeutic drugs than 2D cultures. This *in vitro* difference must be considered in future approaches for determining the ideal *in vitro* systems that mimic human disease.

## Introduction

Colorectal cancer is the third most common cancer in the world, with about 30% of the estimated new cases arising in the rectum [1]. The anatomical localization in the pelvis, as well as its particular blood and lymphatic supply, characterises rectal cancer as a distinct entity with regard to its invasive growth patterns, surgical approach and treatment outcomes [2]. According to current international guidelines, patients with advanced rectal cancer receive either long-course neoadjuvant radiochemotherapy or short-course radiation therapy before complete tumour removal [3]. Neoadjuvant radiochemotherapy or neoadjuvant radiotherapy before surgery is associated with a reduced incidence of local recurrence compared with surgery alone [4–6]. The effect of neoadjuvant therapy on overall survival has been thoroughly debated over the years, but its effectiveness has never been proven. In addition to the improvement of pretreatment regimens, there has also been a major breakthrough in surgical treatment with the introduction of total mesorectal excision (TME) [7].

Despite improved therapy regimens, overall cure rates have changed little and, most importantly, the individual patient response to radiochemotherapy or irradiation therapy alone varies greatly with no possibility of prediction. It is therefore of particular clinical importance to implement new models for predicting the individual response to radiotherapy.

To investigate this inter-individual response to therapy, monolayer cell culture is extensively used in many translational approaches, although there is an evident discrepancy between the results obtained in 2D and 3D cell cultures in terms of drug responses [8–14]. It is speculated that 3D cultures may better reflect a tumour’s *in vivo* situation owing to similarities in oxygen distribution, pH, nutrients, growth factors, cell signalling, and cell matrix organisation [15]. In addition, unlike 2D cultured cells, 3D cultured cells regain their original phenotype and functional activity [16], and are useful models for investigating cellular responses to irradiation therapy on a molecular level [17]. Furthermore, patient-derived organoids can be used to investigate patient response to irradiation therapy [18,19]. However, only limited knowledge has so far been gained on the differences between 2D and 3D cultures in response to irradiation and chemotherapy in a clinically relevant system. The development of an appropriate and clinically relevant *in vitro* model is of particular relevance for the investigation and prediction of the patient’s response to irradiation and chemotherapeutic treatment because 2D cultures are still extensively used to answer these clinical questions.

Here, we aim to investigate the differences in responses to irradiation therapy between 2D and 3D cultures using a clinical Elekta Versa HD accelerator, and responses to chemotherapeutic treatment with doxorubicin, 5-FU, mitomycin C, and cisplatin to determine the ideal *in vitro* system for future research on human disease.

## Material and methods

### Cell culture

Colorectal cancer cell lines (CaCo2, Colo205, HCT116, SW480) were obtained from Jens Schmid (Dr. Margarete Fischer-Bosch Institute for Clinical Pharmacology, Stuttgart, Germany). CRC cell lines were authenticated by STR analysis (Eurofins, Ebersberg, Germany). For quality control, a mycoplasma PCR using the Venor®GeM Classic kit (11–1025, Minerva Biolabs, Berlin, Germany) was performed according to the manual and before the use of cell lines. Cells were either grown as monolayers or seeded and grown as spheroids in 48-well plates. For spheroid cultivation, cells were seeded in drops of matrigel (Corning® Matrigel® Growth Factor Reduced Basement Membrane Matrix, Phenol Red-free, LDEV-free, Corning B.V., New York, US) in RPMI medium (without Glutamine, Biochrom AG, Berlin, Germany) supplemented with 10% FCS (Thermo Fisher Scientific GmbH, Waltham, Massachusetts, US), 1% penicillin–strepotomycin (Biochrom AG, Berlin, Germany), and 1% L-glutamine (Biochrom AG, Berlin, Germany) in a 5% CO_2_ incubator at 37°C. Spheroid growth was monitored daily under a microscope (Olympus CKX41) and spheroids were split when the drops reached confluency. Spheroids were harvested, pelleted, and broken up by incubation with TrypLE™ Express Enzyme (1X, phenol red, Thermo Fisher Scientific GmbH, Waltham, Massachusetts, US), pelleted, split, and seeded in new matrigel drops. Confluency levels for optimal and comparable culture conditions were 30–40% at seeding, 50% at time of treatment, and 80% at time of harvesting for 2D and 3D cultures.

### Irradiation

Colorectal cancer cell lines were grown as monolayers or spheroids in 35-mm dishes and cells were grown for 4–7 days. Afterwards, cells were irradiated with 1, 4, and 10 Gy using an Elekta Versa HD Linear Accelerator (6 MV, gantryi angle 0°, fieldsize 25 cm × 25 cm, focus-to-skin distance of 95 cm). Cell growth was monitored daily under a microscope (Olympus CKX41) using bright field imaging with 10× magnification. Images were obtained before irradiation and 4–6 days after irradiation using an F-View Soft Imaging System.

### Flow cytometry

For analysis of apoptosis rates, cells were harvested 6 days after irradiation as described before. To ensure proper staining, spheroids were broken up by incubation with TrypLE™ Express Enzyme (1X, phenol red, Thermo Fisher Scientific GmbH, Waltham, Massachusetts, US). After dissociation into single cells, the supernatant was removed and collected. Single cells were washed with PBS and resuspended in 1× annexin binding buffer (10×, 0.1 M hepes (pH 7.4), 1.4 M NaCl, 25 mM CaCl_2_) and incubated for 5 min at room temperature. Afterwards, cells were pelleted and stained with annexin V-FITC (Miltenyi Biotech, Bergisch Gladbach, Germany) and propidium iodide solution (Miltenyi Biotech, Bergisch Gladbach, Germany) according to the manufacturer’s manual. Stained cells were analysed by flow cytometry using a FACSLyric system (BD Biosciences, Franklin Lakes, New Jersey, US).

### Immunohistochemistry

Cells were harvested carefully to ensure they retained their spheroid structure, pellets were resuspended in HistoGel (Thermo Scientific, Waltham, Massachusetts, US), fixed with 4% PFA (VWR International, Radnor, Pennsylvania, US), and embedded in paraffin. Sections of 4 μm were stained with haematoxylin (Mayer’s, Sigma-Aldrich Chemie, St. Louis, Missouri, US) and eosin (Merck Chemicals GmbH, Darmstadt, Germany) or Ki-67 (Clone SP6, monoclonal rabbit IgG, Order-no. 275R-16, LOT-no. 0000075544, Cell Marque, Rocklin, California, US). After heat-induced antigen retrieval at pH 9, anti-Ki-67 (1:100) was incubated for 30 minutes. For quantification of apoptosis, TUNEL assay (ApopTag® Plus Peroxidase In Situ Apoptosis Kit, Merck Chemicals GmbH, Darmstadt, Germany) was performed according to the manufacturer’s manual. For each experiment, at least 5 fields of view (20× magnification) per dose were counted and only immunoreactivity in nuclei or apoptotic bodies was considered positive.

### Chemotherapeutic treatment and analysis of cell viability

Chemotherapeutic drugs were obtained from the in-house clinical pharmacy department, and were 2 mg/ml doxorubicin-HCl solution (06581630, Teva GmbH, Ulm, Germany), 50 mg/ml 5-FU solution (10309023, Accord, Munich, Germany), 1 mg/ml mitomycin C solution (11213532, medac, Wedel, Germany), and 1 mg/ml cisplatin solution (06559665, Teva GmbH, Ulm, Germany). Working concentrations, diluted in culture medium, were prepared fresh before use, and cells/spheroids were treated with increasing concentrations for 72 h. Cell viability was assessed using a RealTime-Glo™ MT Cell Viability Assay (Promega, Madison, Wisconsin, US) according to the manufacturer’s manual.

### Gel electrophoresis and immunoblotting

Cells were harvested and washed twice with PBS. For 3D cultures, matrigel was removed from the cells and taken as an internal control. Pellets were resuspended in lysis buffer (50mM Tris-HCl pH 7.6, 250mM NaCl, 5mM EDTA, 0.1% Triton x-100) containing cOmplete protease inhibitor cocktail (4693124001, Sigma-Aldrich Chemie GmbH, Schnelldorf, Germany) and PhosSTOP phosphatase inhibitor (4906845001, Sigma-Aldrich Chemie GmbH, Schnelldorf, Germany). Cells were broken up by two cycles of ultrasound treatment for 20 sec each and protein measurement was performed using Pierce™ BCA Protein Assay Kit (23227, Thermo Fisher Scientific GmbH, Waltham, Massachusetts, US) according to the manufacturer’s manual. 15–20μg protein were separated by SDS-PAGE and transferred onto nitrocellulose membranes (0,45μm). For immunoblotting, membranes were probed with primary antibodies against γH2AX (Ser139, Clone 20E3, monoclonal rabbit IgG, Order-no. 9718, LOT-no. 17, Cell Signaling Technology, Danvers, Massachusetts, US), Chk1 (Clone 2G1D5, monoclonal mouse IgG, Order-no. 2360, LOT-no. 1, Cell Signaling Technology, Danvers, Massachusetts, US), pChk1 (Ser 345, Clone 133D3, monoclonal rabbit IgG, Order-no. 2348, LOT-no. 18, Cell Signaling Technology, Danvers, Massachusetts, US), and β-actin (Clone AC-15, monoclonal mouse IgG, Order-no. A5441, LOT-no. 029M4883V, Sigma-Aldrich Chemie GmbH, Schnelldorf, Germany) overnight at 4°C, then incubated with anti-rabbit IgG-HRP (Order-no. 7074 S, LOT-no. 28, Cell Signaling Technology, Danvers, Massachusetts, US) or anti-mouse IgG-HRP (Order-no. 7076, LOT-no. 33, Cell Signaling Technology, Danvers, Massachusetts, US) for one hour at room temperature. Antibodies were used at a dilution of 1:1000, except anti-β-actin (loading control; 1:5000) and secondary antibodies (1:5000). Signals were detected by enhanced chemiluminescence (SuperSignal West Dura Extended Duration Substrate, 34075, Thermo Fisher Scientific GmbH, Waltham, Massachusetts, US) using CCD camera STELLA3200 (Raytest, Straubenhardt, Germany).

### Statistics

Statistical analyses comparing control and treatment conditions were performed using a two-sided Student’s T test (paired/ unpaired as indicated in the figure legend) in Microsoft Excel 2016. Confidence intervals were calculated using Microsoft Excel 2016 with α = 0.05. All experiments are shown as mean and standard error (SE) of at least three independent experiments.

## Results

In this current work, we compared the response of 2D colorectal cancer (CRC) cell lines and 3D CRC cell-line-derived spheroids to irradiation and chemotherapy. The 2D and 3D CRC cell line cultures (CaCo2, Colo205, HCT116, and SW480) were irradiated with 1, 4, or 10 Gy using a clinical grade linear accelerator (Elekta Versa™ HD). The response to irradiation therapy was analysed by immunohistochemistry, flow cytometry, and TUNEL assays. In addition, 2D and 3D cultures were treated with standard chemotherapeutic drugs and the response to treatment was analysed by a RealTime-Glo™ MT Cell Viability Assay.

### Spheroid integrity is lost after irradiation therapy

**Fig 1:**
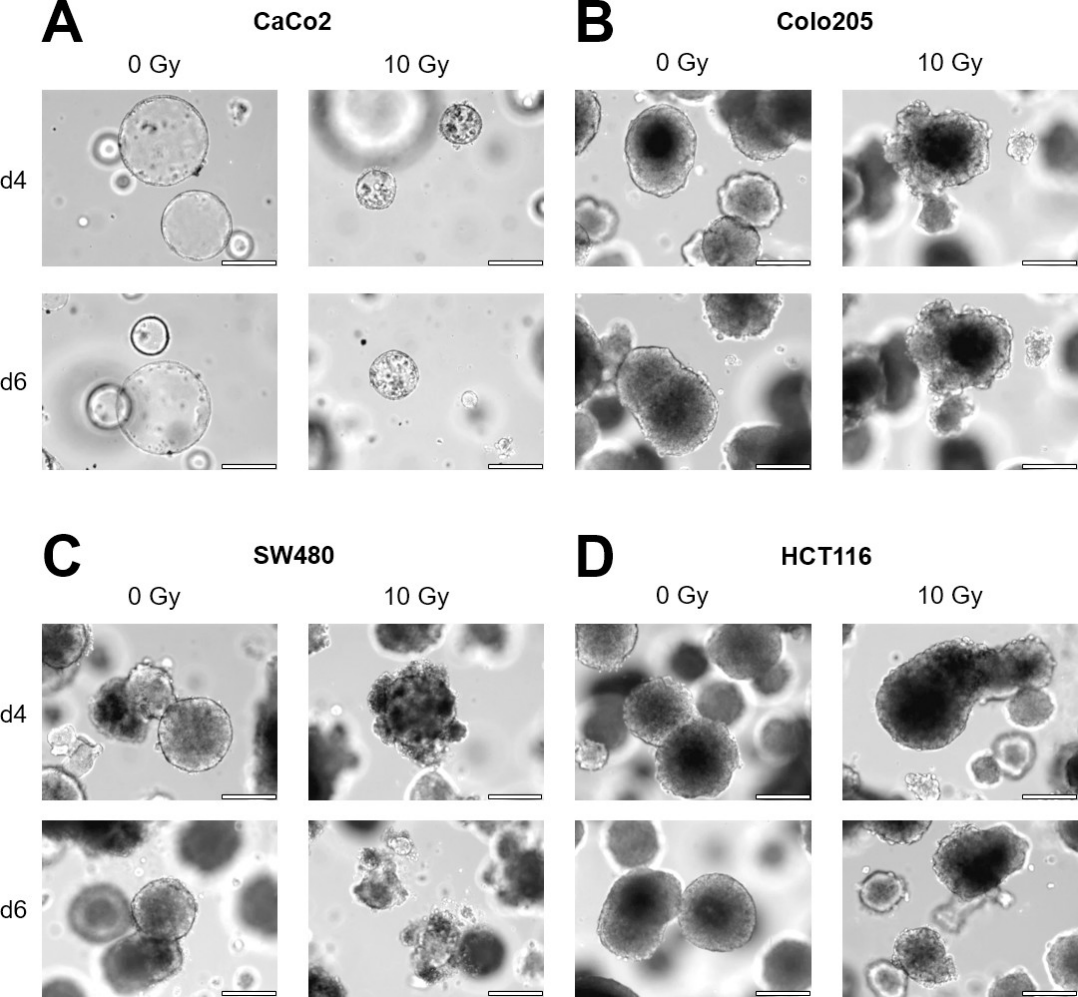
Spheroid integrity is lost after irradiation therapy. CRC cell lines were grown in matrigel as 3D spheroids and irradiated with an Elekta Versa HD accelerator. **(A)** CaCo2 cells form sharp demarcated and perfect spherical structures. **(B-D)** Colo205, SW480, or HCT116 spheroids showed a granular, grape-like structure. Six days after irradiation all spheroids showed a loss of compact morphology, membrane fringing and apoptotic vesicles (scale bar 200μm).

Before treatment, spheroids were analysed by light microscopy, which showed strong morphological and structural differences between the different CRC cell lines; although spheroids derived from CaCo2 cells showed sharply demarcated and perfectly spherical structures (Fig 1A), spheroids derived from Colo205, SW480, or HCT116 cells demonstrated a granular and grape-like structure (Fig 1B–1D). Next, spheroids were irradiated with doses of 1, 4, or 10 Gy using a clinical grade linear accelerator and morphological changes after irradiation were monitored under a microscope. At 4 to 6 days after irradiation, spheroids showed clear morphological changes and signs of apoptosis, which was especially visible for spheroids irradiated with 4 and 10 Gy; morphological changes included reduced spheroid growth, loss of compact structure with the outer membrane and the inner core becoming looser (Fig 1A), and membrane fringing (Fig 1B–1D). Moreover, apoptotic vesicles became visible at the outer membranes, especially at 10 Gy.

### Histological analysis confirms loss of integrity and proliferation

To further analyse and characterise the response of CRC spheroids to irradiation therapy, haematoxylin–eosin (H&E) and immunohistological (IHC) staining of formalin-fixed paraffin-embedded (FFPE) spheroids was performed 6 days after irradiation.

**Fig 2:**
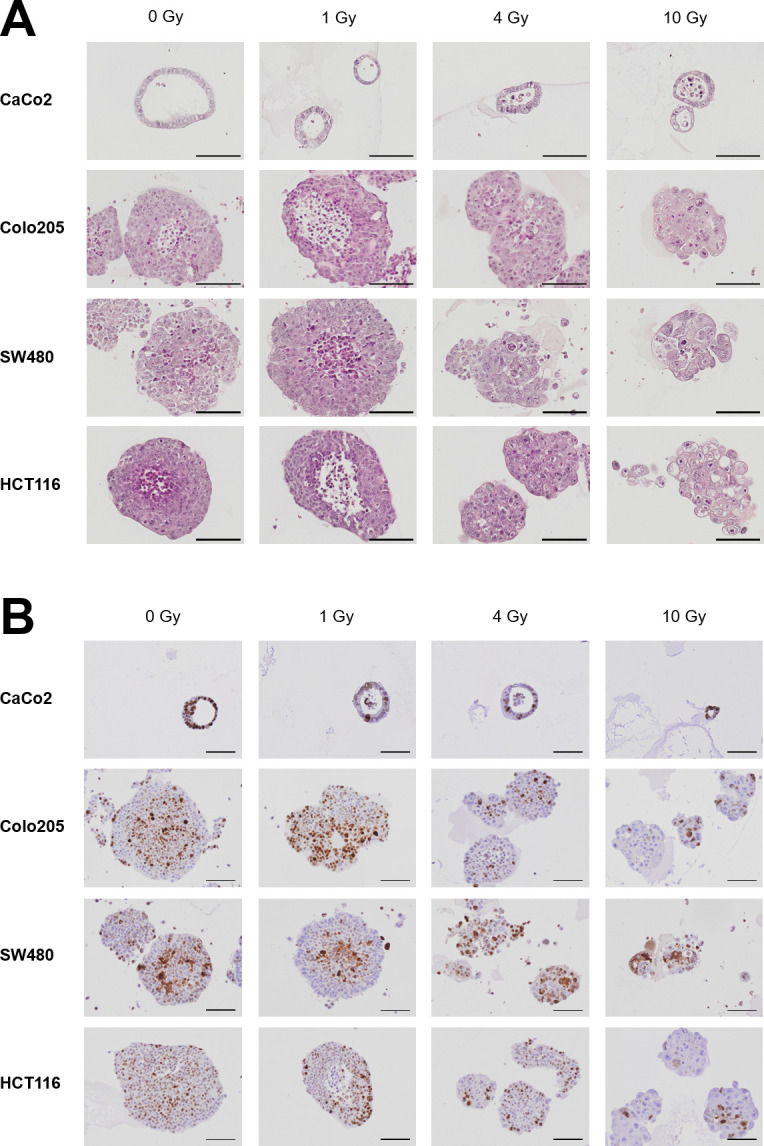
Histological analyses confirm loss of integrity and proliferation. CRC cell lines were grown in matrigel as 3D spheroids and irradiated with an Elekta Versa HD accelerator. Six days after irradiation, spheroids were fixed with 4% PFA, embedded in paraffin, and stained with **(A)** H&E or **(B)** Ki-67 (scale bar 100μm).

Similar to microscopical analyses, H&E staining of CaCo2 spheroids revealed ring-like and sharply demarcated spherical structures with an inner core that was sometimes filled with cells (Fig 2A). In contrast, Colo205, SW480, and HCT116 spheroids appeared with more irregular shapes and granular structures. However, upon irradiation, the spheroids lost their compact structure (especially HCT116) as evidenced by an increase in isolated single cells and smaller spheroids. In addition, swelling of the cell nucleus and membranes as well as nuclear pyknosis/blebbing was observed, especially at 10 Gy, this indicated apoptotic and/or necrotic cell death.

In the next step, proliferation after irradiation was analysed by Ki-67 staining (Fig 2B). Proliferation of CRC spheroids was reduced upon irradiation, as evident by reduced Ki-67 expression, which was especially obvious for the Colo205 and HCT116 spheroids. Moreover, the staining pattern changed upon irradiation from an intense and dark brown staining in the controls to a more diffuse pattern in irradiated cells.

### 3D spheroids show decreased apoptosis rates after irradiation compared with 2D cultures

**Fig 3:**
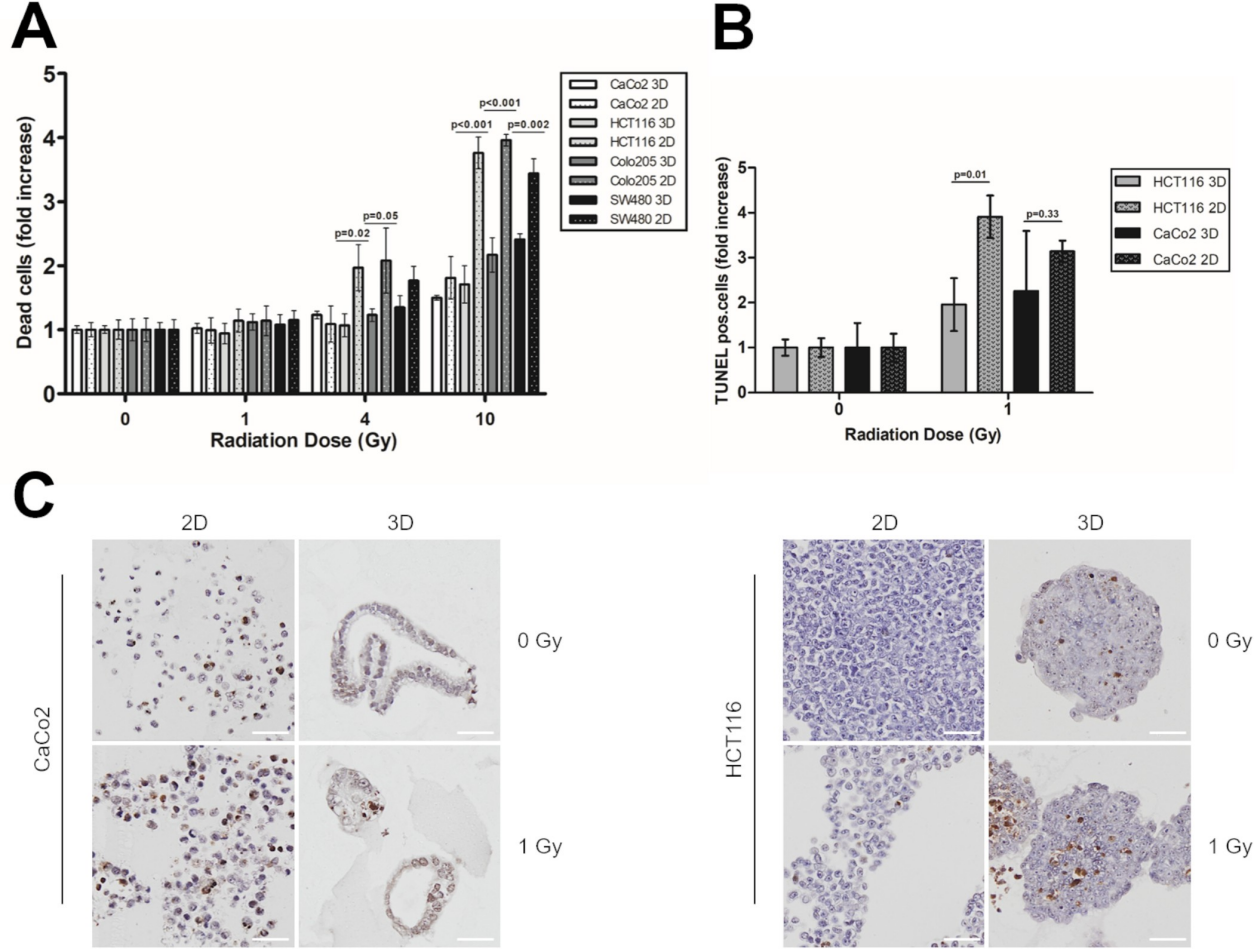
3D spheroids show decreased apoptosis rates after irradiation compared with 2D cultures. **(A)** CRC cells were irradiated with 1, 4, and 10 Gy. Six days after irradiation, cells were stained with annexin V/ PI, and analysed by flow cytometry. Significance was calculated using a two-sided, unpaired Student’s T test. **(B)** CaCo2 and HCT116 cells were irradiated with 0 and 1 Gy. Six days after irradiation, cells were fixed with 4% PFA, embedded in paraffin and stained for TUNEL assay. Significance was calculated using a two-sided, unpaired Student’s T test. **(C)** Representative TUNEL stainings for CaCo2 and HCT116 2D and 3D cultures six days after irradiation with 0 and 1 Gy. Scale bar 50 μm.

In a next step, irradiated cells from 2D and 3D cultures were incubated for 6 days and analysed by flow cytometry using annexin V/PI staining (Fig 3A and S1 Raw images). Upon irradiation with 10 Gy, and compared with non-irradiated cells, 2D cell cultures revealed an 1.8–4-fold (95% CI 3.24±0.96) increase in apoptosis rates, and 3D spheroids showed an 1.5–2.4-fold increase (95% CI 1.56±0.41). This indicated that 2D cells were more sensitive to irradiation therapy than 3D spheroids. These results were then confirmed by TUNEL assays (Fig 3B and 3C). Following irradiation with 1 Gy, CaCo2 and HCT116 3D spheroid cells showed a 2-fold increase in TUNEL positive cells, compared with a 3- (p = 0.33), and 4-fold (p = 0.01) increase, respectively, for the 2D cultures. Thus, compared with 3D spheroids from the same cell line, 2D monolayer cultures were more sensitive to irradiation. To analyse whether increased apoptosis rates were caused by increased DNA damage, we performed γH2AX staining six days after irradiation in 2D and 3D cultures (data not shown). However, we could not detect any differences between the different culture conditions, which might be attributed to the late time point chosen.

### 2D and 3D cultures respond differently to chemotherapeutic drugs

**Fig 4:**
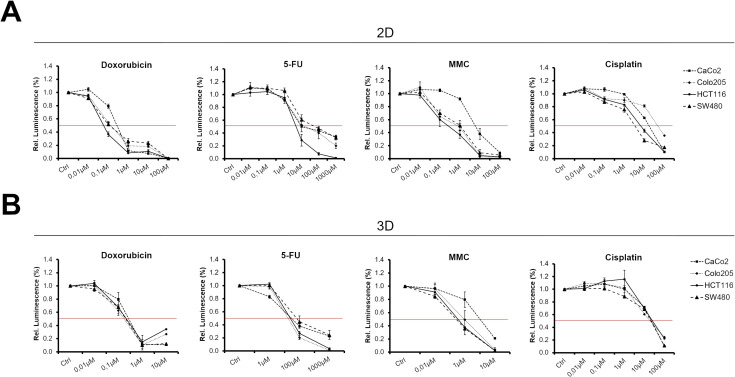
2D and 3D cultures respond differently to chemotherapeutic drugs. **(A)** 2D and **(B)** 3D CRC cultures were treated with the indicated chemotherapeutics for 72h. Afterwards, cell viability was analysed by RT-Glo assay. The red line indicates IC_50_ thresholds.

Next, we investigated whether 2D and 3D cultures of CRC cell lines responded differently to chemotherapeutic treatment. For this, 2D monolayers were treated with increasing concentrations of doxorubicin, 5-FU, mitomycin C, or cisplatin (Fig 4A and Table 1). For 2D cultures, IC_50_ values for doxorubicin and 5-FU ranged between 0.075–0.4 μM and 7.5–75 μM, respectively. IC_50_ values for mitomycin C were 0.5–1 μM for Colo205, HCT116, and SW480. However, with an IC_50_ of 7.5 μM, CaCo2 cells were more resistant to mitomycin C. For cisplatin, IC_50_ values for 2D cultures ranged from 5–75 μM.

**Table 1:** IC_50_ values of different chemotherapeutic drugs in 2D and 3D CRC cultures.

Cell line	IC_50_ value 2D [μM]	IC_50_ value 3D [μM]
**Doxorubicin**		
CaCo2	0,40	0,40
Colo205	0,20	0,25
HCT116	0,08	0,30
SW480	0,10	0,30
averaged IC_50_ (p = 0,121)	0,19	0,31
**5-FU**		
CaCo2	10,00	75,00
Colo205	10,00	60,00
HCT116	7,50	75,00
SW480	75,00	90,00
averaged IC_50_ (p = 0,027)	25,63	75,00
**MMC**		
CaCo2	7,50	5,00
Colo205	1,00	1,00
HCT116	0,50	0,50
SW480	1,00	0,50
averaged IC_50_ (p = 0,297)	2,50	1,75
**Cisplatin**		
CaCo2	25,00	30,00
Colo205	75,00	30,00
HCT116	7,50	40,00
SW480	5,00	30,00
averaged IC_50_ (p = 0,818)	28,13	32,50

Averaged IC_50_ values are means of values from all four cell lines. Significance between means of 2D and 3D cultures was calculated using a two-sided, paired Student’s T test.

Afterwards, 3D spheroids were tested in response to chemotherapeutic treatment (Fig 4B and Table 1). IC_50_ values for doxorubicin were between 0.25 and 0.4 μM, which was similar to the values obtained for the 2D cultures (p = 0.121 for averaged IC_50_ values). With IC_50_ values of 60–90 μM, 3D spheroids, especially HCT116 derived spheroids, were significantly more resistant to 5-FU than 2D cultures (p = 0.027 for averaged IC_50_ values). For mitomycin C, IC_50_ values for spheroids from the Colo205, SW480, and HCT116 cell lines were between 0.5–1 μM and 5 μM for CaCo2 derived spheroids. Thus, IC_50_ values for mitomycin C were in the same range as those of the 2D cultures (p = 0.297 for averaged IC_50_ values). For cisplatin, IC_50_ values of the 3D cultures were 30–40 μM, which were higher for HCT116 and SW480 derived spheroids than their 2D counterparts (p = 0.818 for averaged IC_50_ values). Thus, the 2D and 3D CRC cell line cultures responded similarly to doxorubicin and mitomycin C; however, the 3D cultures were more resistant to cisplatin and especially to 5-FU than the 2D cultures.

**Fig 5:**
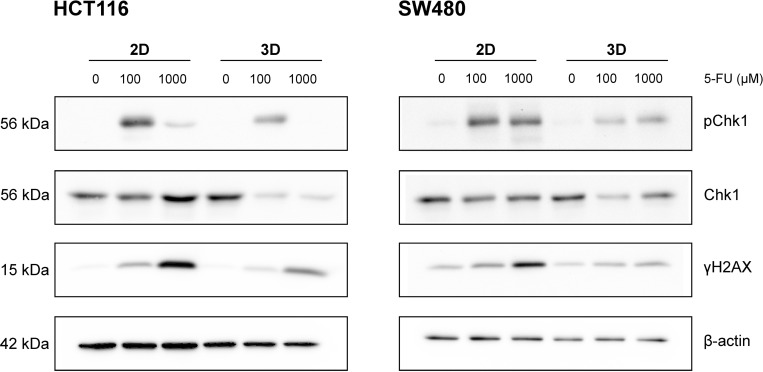
Upon 5-FU treatment the DNA repair pathway is activated stronger in 2D cultures. 2D and 3D cultures of HCT116 and SW480 cell lines were treated with 5-FU at the indicated concentrations for 24h. Afterwards, cells were harvested and analysed by immunoblotting. The size of the proteins is shown on the left. Figure shows representative blots from three independent experiments.

Next, we investigated the expression of proteins of the DNA damage and repair pathways in response to 5-FU treatment in 2D and 3D CRC cultures. Therefore, we analysed HCT116 and SW480 as these cell lines showed the strongest and weakest differences in IC_50_ values for 5-FU between 2D and 3D cultures (Table 1). As shown in Fig 5, γH2AX and pChk1 were upregulated upon increasing 5-FU concentrations in 2D and in 3D cultures and in both cell lines tested. However, activation of γH2AX and pChk1 was stronger in 2D than in 3D cultures, which is in line with the increased IC_50_ value of 3D cultures. As cancer stem cells are known to be more resistant to cytotoxic treatment, we also investigated the expression of the stem cell marker CD44 in 2D and 3D cultures and in response to 5-FU treatment. However, we did not detect any significant CD44 expression, neither in 2D nor in 3D cultures (data not shown).

## Discussion

In this study, we compared the response of 2D CRC cell lines and 3D CRC cell-line-derived spheroids to irradiation and chemotherapy, showing that the response to therapy differed between the 2D and 3D cultures. This is of great importance because most studies are still performed with 2D cultures. However, if such results are transferred to the clinical setting, it will become necessary to work with models that reflect the *in vivo* situation as close as possible.

Previous studies investigated the difference in response to irradiation therapy between various spheroids or patient-derived organoids, showing that there do exist differences between various spheroids and organoids [17–20]. However, to our knowledge, no study has so far investigated the differences in response to irradiation therapy between cell-line derived 2D and 3D cultures in a clinically relevant setting. This is important because most studies investigating therapy responses still use common 2D cultures, which probably do not reflect the real *in vivo* situation. A previous study by Al-Ramadan et al. showed that it was feasible to use multicellular spheroids in combination with IHC analyses to unravel radiobiological responses at a molecular level [17]. However, the authors did not compare their results to 2D cultures or patient outcomes. Another study by Nakajima et al. showed that different patient-derived spheroids of small cell cervical carcinomas responded differently to irradiation therapy and that the response correlated well with the response of engrafted *in vivo* xenografts [20]. Other studies investigated whether the response of patient-derived organoids was comparable to patient outcomes after chemoradiation therapy [18,19]; in those studies, the responses of patient-derived organoids correlated with patient outcomes and predicted the response of rectal cancer patients in a clinical setting. Although, a direct comparison between the drug concentrations used for patient-derived organoids and patients is generally difficult, correlations between sensitive and resistant organoids and primary tumours/metastases can still be made. In this study, we used irradiation doses and drug concentrations that were already previously described by others [18].

In addition, previous studies showed that differences exist between 2D and 3D cultures, primarily in response to drug treatment [8–13,21]. Chitocholtan et al. showed that different endometrial cancer cell lines formed spheroids with distinct 3D morphologies and that those 3D spheroids were more resistant to doxorubicin treatment than 2D monolayers [10]. However, in the present study, we could not observe any differences between 2D and 3D cultures in response to doxorubicin treatment. Discrepancies between our study and the study by Chitocholtan et al. may be explained by the different cancer cell lines used. In the present study, 3D spheroids were more resistant to 5-FU and cisplatin than their 2D counterparts. This was confirmed by immunoblotting, which showed an upregulation of DNA damage repair pathway proteins upon 5-FU treatment in 2D and 3D cultures. However, activation of γH2AX and pChk1 was stronger in 2D than in 3D cultures of both HCT116 and SW480. These differences in sensitivity to cytostatic treatment may be explained by the dense 3D structure of spheroids, especially CaCo2 spheroids, making them more resistant to treatment than their 2D counterparts [11]. It seems that spheroids with dense 3D structures, such as CaCo2 spheroids, were more resistant to treatment, while cell lines that formed loose 3D spheroids tended to have similar sensitivities in the 2D and 3D cultures in the study by Chitocholtan et al. [11]. It is possible that, owing to their dense 3D structure, compact spheroids were more resistant to drug treatment than looser spheroids as the drug could not diffuse equally to all regions of the spheroids, especially the centre. In addition, big spheroids such as those derived from Colo205 and HCT116 cell lines revealed looser, necrotic regions in the centre (Fig 2A) indicating the presence of a heterogeneous cell population within the spheroids [11,22]. It is also possible that drug treatment induces changes in the cell-cell interaction of cells located at the spheroid rim, enableing an increased penetration of nutrients to the spheroid centre thereby initiating cell proliferation of quiescent cells [11].

In addition, it is known that the cellular microenvironment, including matrigel, of multicellular structures can regulate integrin, gene and protein expressions, which differ from their 2D counterparts [11,23,24]. As matrigel is commonly used to cultivate 3D spheroids, it could stimulate cell aggregates to form compact spheroids thereby interfering with cytoskeletal organisation and cell aggregation and adhesion proteins such as E-cadherin and integrins [11,24]. In sum, these changes within and between cells could account for the differences observed between 2D and 3D cultures.

Moreover, it is known that tumour-surrounding cells, including fibroblasts and immune cells, have an impact on tumour cells- a point that is usually not considered in 2D cultures. However, adding different cell types and thus heterogeneity to 3D models, would also add complexity which would make it difficult to answer the question whether culturing conditions (2D vs 3D) only contribute to tumour cell resistance. Therefore, we here decided to focus on culturing conditions only (2D vs. 3D) to investigated the contribution of culture conditions on tumour cell resistance [11,23,24]. Thus, if irradiation and chemotherapeutic results are transferred to the clinical setting, it will become necessary to work with models that are as close to the patient’s situation as possible. Currently, the response to irradiation therapy is difficult to predict and, at present, there exists no marker to identify responding and non-responding patients; one of the most hopeful prospect is Ki-67, which was shown to be strongly expressed in responding tumours [25,26]. However, none of these markers has so far become clinically routine.

In this study, 3D CRC cell line cultures were determined to be more resistant to irradiation and chemotherapy than their 2D counterparts. Further research is in progress to demonstrate whether 3D cultured cells, especially patient-derived organoids, better reflect *in vivo* tumours in patients and whether these results are comparable to patient responses. Therefore, this setup will be used to predict patient response and to identify patients who might respond to irradiation therapy and those who would probably not respond.

## Supporting information

S1 Raw images Gating strategy for flow cytometry analysis. Colo205 cells were irradiated with 0 Gy **(A)** and 10 Gy **(B)**. After 6 days, cells were harvested, stained for annexin V/PI and analysed by flow cytometry. Left blot: x-axis: annexin V-FITC; y-axis: PI.
(TIF)

S2 Raw images (TIF)
