# Supplementary figures and images for: Three dimensional cultivation increases chemo- and radioresistance of colorectal cancer cell lines
Source: PLoS One. 2021 Jan 4;16(1):e0244513. doi: 10.1371/journal.pone.0244513 (PMC7781370; doi:10.1371/journal.pone.0244513)

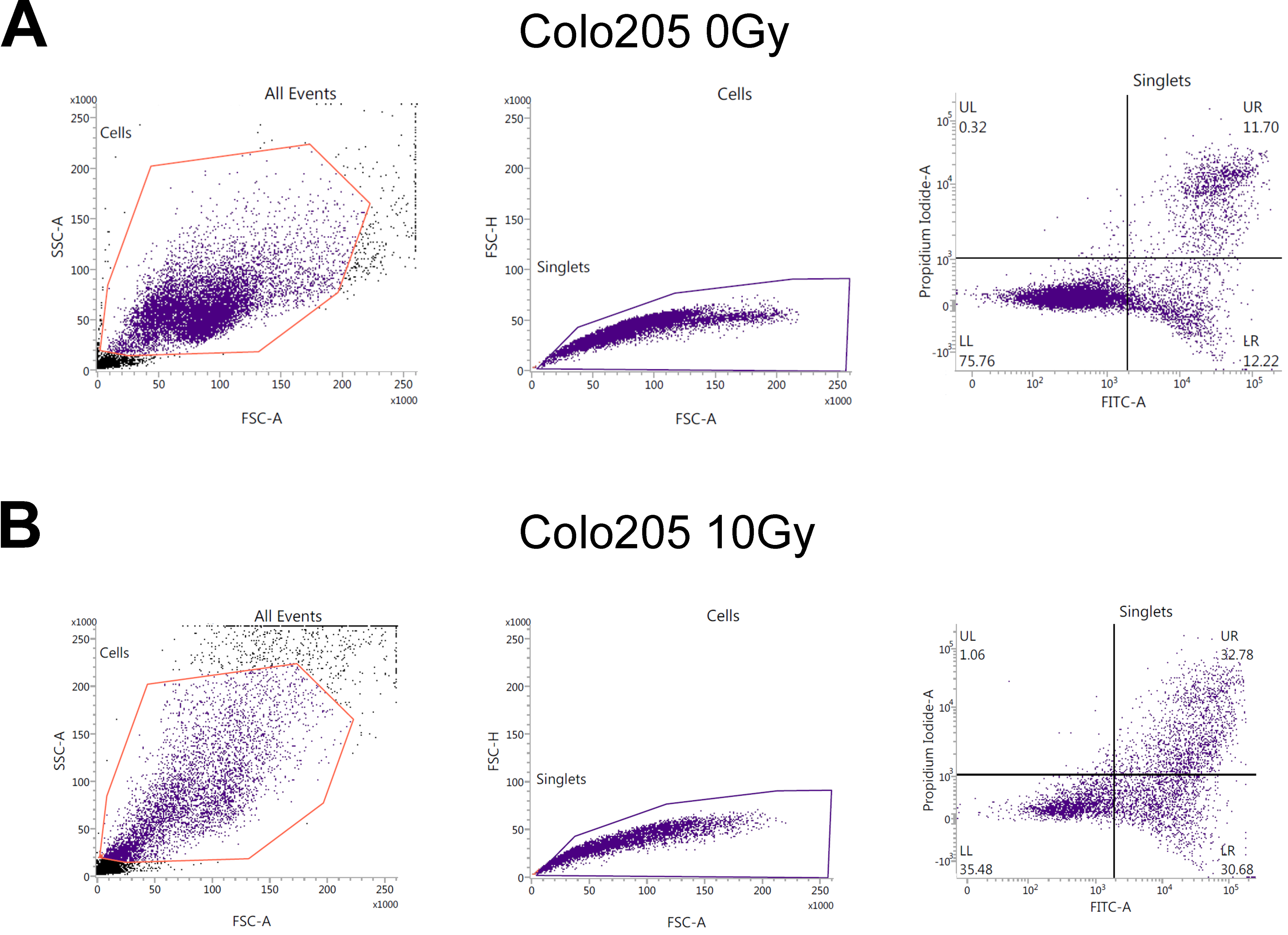

Supplement: S1 Raw images — Colo205 cells were irradiated with 0 Gy (A) and 10 Gy (B). After 6 days, cells were harvested, stained for annexin V/PI and analysed by flow cytometry. Left blot: x-axis: annexin V-FITC; y-axis: PI. (TIF) [file pone.0244513.s001.tif]

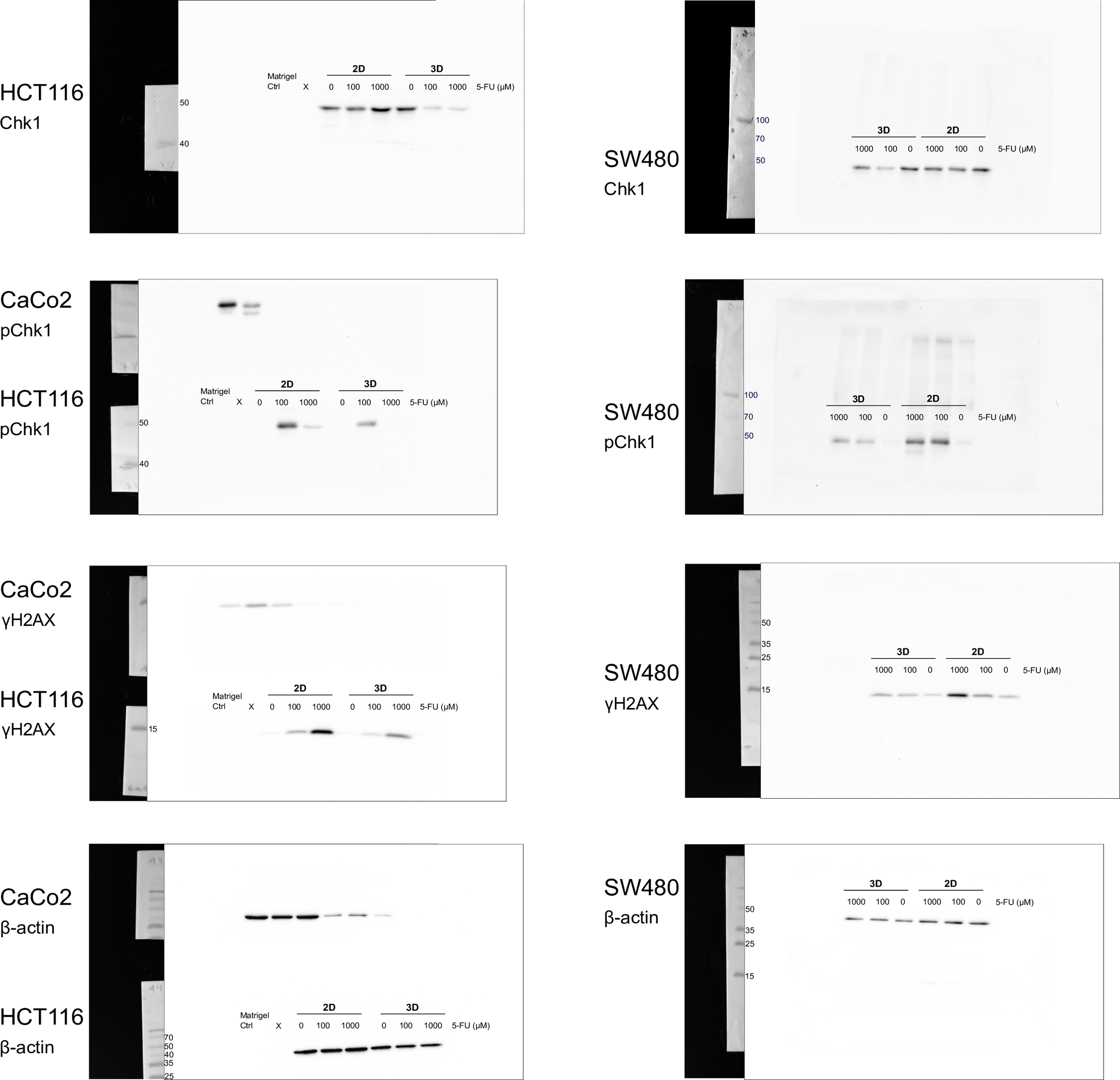

Supplement: S2 Raw images — (TIF) [file pone.0244513.s002.tif]
